# Two Cases of Restlessness—II

**Published:** 1908-04-18

**Authors:** Charles Mercier

**Affiliations:** Physician for Mental Diseases at Charing Cross Hospital.


### Mental Diseases.

### TWO CASES OF RESTLESSNESS—II.

By CHARLES MERCIER, M.D., F.R.C.P., Physician for Mental Diseases at Charing Cross Hospital.

The second case of restlessness was of a somewhat different nature, but it agreed with the first in disclosing on investigation a much graver state of affairs than appeared from the complaint of the patient.

C. D., a clerk, *æt.* 35, was knocked down by the open door of a train that came into the station as he was standing on the platform too near the edge. He was struck in the face, and was insensible for about ten minutes. The next day he went to business, but was obliged to return home, being unable to work. He became giddy, and everything seemed blurred. He was in bed for three weeks, and then went away for a week. Since attempting to resume work he has found himself unable to do so on account of "restlessness." He cannot settle to anything. He gets so restless that he cannot sit over his work for more than half an hour; then he has to get up and go out. Since the accident he has been unable to sleep for more than an hour or two every night, though formerly he was a very good sleeper. He suffers also from a feeling of tightness in the head, which is relieved by gripping something with his hands and squeezing it hard. He is very anxious to get back to work, but is unable to work on account of the restlessness and inability to settle down steadily to anything. He often wants to cry.

As I put a question to him, his gaze became fixed and his eyes expressionless; he stared fixedly at a corner of the room; his face became grey; he did not answer. Then he drew himself slowly back in his chair, his eyelids quivering; and at the end of about half a minute, he roused up, his face flushed, and he turned to me saying, "What question was that?" Then he put his face in his hands and began to cry. He said it was "the feeling" again. He often had these feelings, and when a "feeling" was over he wanted to hurt someone. The day before he had given his little boy a thrashing. The boy had told his mother a lie, and would have been punished in any case, but "the horrible part of it was that I took a delight in thrashing him; I felt that I wanted to hurt him." These "feelings" were always followed by the same desire to hurt some one. The other day he got hold of a cat and nearly killed it. The cat had not offended him in any way. The only means of combating this desire to hurt things is to get up and go out of the house, and then it passes away in a few minutes. This is the secret of his restlessness. The attacks are frequent, and whenever he has one he is impelled—he finds it necessary—to get up and go out of the house, so as to "work it off," and thereby avoid attacking people. The impulse is not to injure anyone in particular; nor is it always, as in the case of the boy and the cat, against something small, nor against any particular class of person or things. After this last "feeling" he felt strongly inclined to attack me. The impulsion is against anything or anyone that happens to be near him.

As I was talking to him he had another attack. His gaze became fixed and very intent, then more and more intent until he assumed an expression of actual ferocity. Next he drew in a slow, deep, shuddering breath; drew himself up and back; his eyes closed with a quivering motion as his face turned grey; then he flushed and began to cry as before, and he was himself again.

The case was clearly one of *petit mal*, in which the post-epileptic automatism of action, which is so customary, is replaced by the automatic occurrence of a desire. I have never come across a similar case, and the lessons that it teaches are many. Post-epileptic automatism is elaborate action, a caricature of normal action, and during the action there is, as far as subsequent recollection is concerned, total unconsciousness. In this case the post-epileptic occurrence is not action, but the experience of a desire which might prompt to action; but if it did, the action would be not an unintelligent caricature of normal action, but action fully directed by intelligence, and fully calculated to secure the satisfaction of the desire. In short, the desire, not the action, would be abnormal. Rare as it is, there is nothing in the replacement of post-epileptic automatic action by a desire that is inconsistent with the currently accepted mechanism of post-epileptic automatism. This is due to the uncontrolled overaction of lower nervous arrangements permitted by the exhaustion of higher arrangements that should, and normally do, control and inhibit the lower. Normally, our predatory and feral desires are controlled and inhibited by social desires of later origin, and therefore of higher rank. If we assume that these social desires are embodied in nervous arrangements capable, like other nervous arrangements, of being exhausted and rendered incompetent by the excessive discharge of epilepsy, there is nothing inconsistent in supposing that, by this exhaustion, arrangements of lower rank, embodying cruder desires of earlier origin, may be set free to overact, with accompanying excessive manifestation of such crude desire. The interesting feature of this case is that it goes far to establish what has never heretofore been anything but an unsupported speculation, that specific desires may be regarded as accompanying the action of specific regions of nerve tissue.

Another very important feature in the case is the light it throws on *epilepsie larvée*. Certain crimes of brutal and ferocious violence are committed by epileptics, not after a fit, but at a time when a fit is due to occur, and seemingly in place of a fit. If we suppose that in such cases a fit does occur, but is of very minor severity, and affects merely the highest arrangements of a particular region, allowing excessive activity of the underlying region; then, if the underlying region so overacting is the region affected, but affected less severely, in this very case, it goes far to explain the occurrence of these crimes, and to justify the hypothesis of *epilepsie larvée*.

